# Chronic Infections and Diabetes in Africa: A Narrative Review of Pathogen-Associated Metabolic Risk

**DOI:** 10.7759/cureus.99923

**Published:** 2025-12-23

**Authors:** Olabisi P Lawal, Fortune I Ebiala, Modinat Abayomi, Obiageri Ihuarulam Okeoma, Nana Ama Aduma Amankwah, John O Patrick, Queeneth Eguakun, Aliyu O Olaniyi

**Affiliations:** 1 Medical Laboratory Science, University of Benin, Benin City, NGA; 2 Medicine/Infectious Diseases/Microbiology, Washington University of Health and Science, San Pedro Town, BLZ; 3 Microbiology/Antimicrobial Resistance/Infectious Diseases, University of Benin, Benin City, NGA; 4 Biology, Boston College, Boston, USA; 5 Medical Laboratory Science, Trinity University Yaba, Lagos, NGA; 6 Health and Wellness Services, Western Illinois University, Macomb, USA; 7 Laboratory/Clinical Chemistry, St. Gerard's Catholic Hospital, Kaduna, NGA; 8 Microbiology, University of Benin, Benin City, NGA; 9 Geriatrics, Stepping Hill Hospital, Stockport, GBR

**Keywords:** chronic infections, diabetes, dysglycaemia, helicobacter pylori, hiv, infectious determinants, metabolic dysfunction, tuberculosis

## Abstract

Diabetes is increasing rapidly across Africa, where individuals in low-income areas are found to develop the disease at younger ages and lower body mass indices than those in high-income regions. Traditional metabolic risk factors explain only part of this trend, prompting growing interest in the role of chronic infections as contributors to dysglycemia. Persistent exposure to pathogens such as HIV, tuberculosis, hepatitis C virus, *Helicobacter pylori*, periodontal bacteria, cytomegalovirus, and parasitic organisms remains widespread in the region. These infections may influence glucose metabolism through sustained low-grade inflammation, immune-mediated pancreatic injury, alterations in gut microbial composition, and treatment-related metabolic effects. This narrative review synthesizes current evidence on the infectious determinants of metabolic dysfunction, with a focus on pathogens of high relevance in African settings. The strongest epidemiological signals link HIV infection and antiretroviral therapy exposure, chronic hepatitis C virus infection, and tuberculosis with impaired glucose regulation. Evidence for *H. pylori*, periodontal disease, and parasitic infections is emerging, but the findings are less consistent. African studies support the plausibility of infection-related metabolic disturbances, although most available investigations are cross-sectional, which limits causal inference. Laboratory and mechanistic research remains sparse, and diagnostic variability further constrains interpretation. The review highlights several clinical implications, including the value of incorporating glycemic screening into HIV and tuberculosis programs, the need to consider metabolic effects when selecting antimicrobial therapies, and the importance of integrating lifestyle counselling into chronic infection care. Strengthening longitudinal research, improving diagnostic standardization, and developing integrated infectious disease-non-communicable disease care models will be essential for addressing the intertwined burdens of infection and diabetes in Africa. Understanding how chronic pathogens interact with metabolic pathways may help refine prevention strategies and promote earlier detection of dysglycemia in high-risk populations.

## Introduction and background

Diabetes is rising rapidly across Africa, where many cases remain undiagnosed until complications occur [1]. Recent estimates indicate that over 24 million adults in Africa are living with diabetes, with projections suggesting this number will more than double by 2045. Notably, approximately 50-60% of cases remain undiagnosed, contributing to delayed presentation and higher complication rates [2].

Although global increases in diabetes are largely explained by ageing, urbanization, and lifestyle transitions, these factors alone do not fully account for patterns seen in many African populations, where diabetes often presents at younger ages and at lower body mass indices compared to high-income regions [3]. Conventional models that emphasize adiposity and sedentary behaviour, therefore, capture only part of the risk in settings where infectious diseases remain widespread [4].

Over the past two decades, growing evidence has highlighted the possibility that chronic or recurrent infections contribute to dysglycaemia through sustained inflammation, disturbances of the gut microbiome, immune-mediated β-cell injury, and the metabolic effects of antimicrobial therapies [4]. Persistent exposure to pathogens is common across many African regions, where HIV, tuberculosis, hepatitis viruses, *Helicobacter pylori*, periodontal disease, and parasitic infections coexist with emerging epidemics of type 2 diabetes. This epidemiological overlap has prompted increasing interest in understanding whether chronic infections may accelerate metabolic dysfunction in susceptible individuals [5].

Persistent exposure to pathogens is common across many African regions, where sub-Saharan Africa accounts for approximately two-thirds of the global HIV burden, and tuberculosis incidence remains among the highest worldwide. Hepatitis C virus seroprevalence exceeds 3-5% in some populations, while *H. pylori* infection affects a majority of adults in several settings [6].

Despite rising attention, the infection-diabetes relationship remains under-recognized in routine clinical practice. Most available evidence comes from cross-sectional studies, and relatively few investigations originate from African settings, where co-infection patterns, nutritional profiles, and health-system constraints differ from those in high-income countries. Clarifying how infectious diseases shape metabolic risk is therefore essential for both clinicians and policymakers.

This review aims to synthesize current knowledge on infection-related metabolic dysregulation, emphasizing pathogens most relevant to Africa. The review summarizes proposed biological mechanisms, evaluates the epidemiological evidence, and highlights practical implications for screening and integrated care in resource-constrained settings. Taken together, the convergence of a rapidly expanding diabetes epidemic with a persistently high burden of chronic infections underscores the need to re-examine conventional metabolic risk models in African populations. 

Methodology

Search Strategy

This narrative review was developed to synthesize existing evidence on the relationship between chronic infections and disturbances in glucose metabolism, with particular emphasis on African settings. A structured literature search was conducted between January and December 2024 using PubMed, Google Scholar, Scopus, and Web of Science. Search terms included combinations of “diabetes”, “dysglycaemia”, “insulin resistance”, “HIV”, “hepatitis C”, “tuberculosis”, “Helicobacter pylori”, “periodontal disease”, “cytomegalovirus”, “schistosomiasis”, “malaria", and “Africa”. Additional relevant publications were identified by screening the reference lists of eligible articles and recent reviews.

The full search strategy combined infection-specific terms with metabolic outcomes using Boolean operators (e.g., “HIV OR tuberculosis OR hepatitis C” AND “diabetes OR dysglycaemia OR insulin resistance”)

Eligibility Criteria

Studies were considered eligible if they examined associations between infectious diseases and glucose metabolism, described biological mechanisms linking infection to metabolic dysfunction, or reported epidemiological data relevant to African populations. Priority was given to peer-reviewed observational studies, cohort analyses, mechanistic investigations, and interventional studies. Articles not published in English were excluded unless an English translation was available.

Study Selection and Data Extraction

Titles and abstracts were screened for relevance, followed by a full-text review of potentially eligible articles. Data extracted from included studies comprised pathogen type, study design, population characteristics, metabolic outcomes, proposed mechanisms, and reported clinical or public health implications. Emphasis was placed on studies conducted in African settings, while key international studies were included where African data were limited. Studies were selected through sequential title and abstract screening followed by full-text review, with relevance assessed based on predefined eligibility criteria and thematic alignment with the review objectives.

Data Synthesis

Due to heterogeneity in study design, diagnostic criteria, and outcome measures, a formal meta-analysis was not undertaken. Instead, findings were synthesized narratively and organized thematically according to mechanistic pathways and pathogen relevance. The review highlights consistent epidemiological patterns, emerging evidence, and gaps in knowledge to inform clinical practice and future research. Ethical approval was not required, as this study involved analysis of previously published data.

Formal risk-of-bias assessment tools were not applied, as this was a narrative review rather than a systematic review. However, the strengths and limitations of the available evidence were considered during synthesis, including study design, consistency of findings, and relevance to African settings. Most epidemiologic studies were cross-sectional, limiting causal inference, and variability in diagnostic criteria and confounder adjustment was common. These factors were taken into account when interpreting the strength of associations described. Formal statistical pooling, meta-analysis, or meta-regression was not performed due to substantial heterogeneity in study designs, populations, infection definitions, and glycaemic outcome measures across the included studies.

## Review

Conceptual framework

Understanding how chronic infections influence glucose metabolism requires consideration of several interconnected biological pathways. Persistent or recurrent pathogens can generate metabolic disturbances through immune, endocrine, microbial, and tissue-specific mechanisms that evolve [7]. Although originally adaptive in acute infection, prolonged activation of these pathways may gradually shift an individual from normal glucose regulation to insulin resistance and β-cell dysfunction [8].

A central mechanism involves sustained low-grade inflammation, which impairs insulin signaling, increases hepatic glucose output, and reduces peripheral glucose uptake. Over time, this inflammatory environment exacerbates oxidative stress and mitochondrial dysfunction, contributing to insulin resistance [9].

A second pathway concerns immune-mediated or pathogen-related injury to pancreatic β-cells. Some infections provoke cytokine cascades that alter β-cell function or induce apoptosis. Others may indirectly stimulate autoimmune activity or enhance pre-existing immune dysregulation, leading to loss of insulin-producing capacity [10].

Alterations in the gut microbiome represent another important mechanism. Chronic infections can disrupt microbial diversity and weaken gut barrier integrity, facilitating the translocation of bacterial products such as lipopolysaccharide into the circulation. These microbial products amplify systemic inflammation and interfere with metabolic signaling networks involved in glucose and lipid homeostasis [11].

A final pathway involves pathogen-derived molecules or infection-related tissue effects that impair insulin signaling or organ function. Viral proteins, bacterial toxins, and helminth-associated metabolites may modify adipocyte behaviour, hepatic insulin sensitivity, or neurohormonal regulation of appetite and energy balance [12].

**Figure 1:**
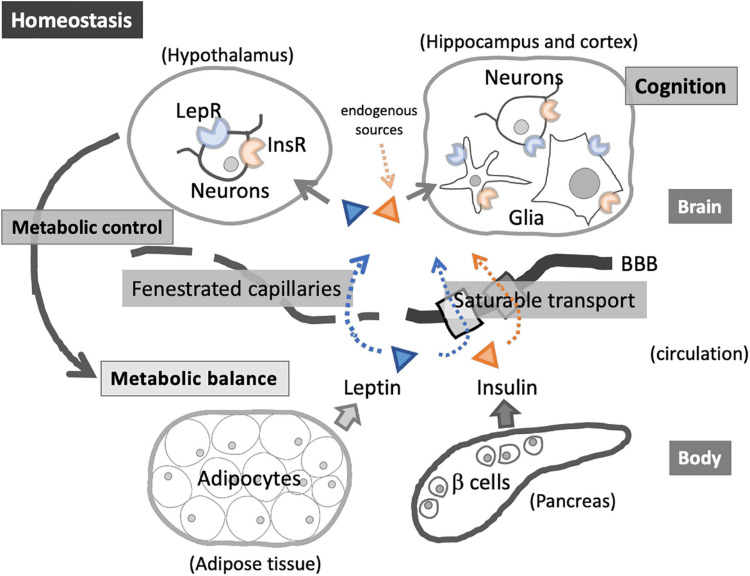
The infection–metabolism axis linking chronic infections to dysglycaemia Conceptual illustration of central and peripheral metabolic regulatory pathways involving insulin and leptin signaling between adipose tissue, pancreas, circulation, and the brain. Chronic or recurrent infections common in African settings may disrupt these pathways through sustained inflammation, immune activation, gut microbiome alterations, and treatment-related metabolic effects, thereby promoting insulin resistance, β-cell dysfunction, and impaired glucose regulation Image Source: Shinjyo and Kita, 2021 [13]; Distributed under the terms of the Creative Commons Attribution (CC BY) CC BY 4.0, Attribution 4.0 International Deed

Together, these processes form an “infection-metabolism axis” through which pathogens and host responses interact to influence glycaemic control (Figure 1). While the relative contribution of each mechanism varies by pathogen and individual susceptibility, the collective effect may increase vulnerability to dysglycaemia in populations with high burdens of chronic or recurrent infections. As illustrated in Figure 1, multiple infectious exposures share convergent biological pathways that can impair glucose regulation [13].

Pathogen-specific evidence

A growing body of evidence links specific chronic infections to disturbances in glucose metabolism, although the strength of association varies by pathogen. In African settings, the most consistent signals arise from HIV infection and antiretroviral therapy exposure, chronic HCV infection, and active or previously treated tuberculosis. Evidence for other pathogens, including Helicobacter pylori, periodontal disease, cytomegalovirus, and selected parasitic infections, is emerging but remains less conclusive [14,15].

HIV and Antiretroviral Therapy

HIV continues to be one of the most influential infectious exposures affecting metabolic health on the continent. Chronic immune activation characteristic of untreated HIV contributes to insulin resistance, adverse lipid profiles, and alterations in fat distribution, while several antiretroviral agents exert additional metabolic effects. Older protease inhibitors and certain nucleoside analogues have well-defined associations with insulin resistance, and more recent data suggest that some integrase inhibitors may induce weight gain in susceptible individuals [16,17]. Large African cohort studies consistently demonstrate a higher prevalence of diabetes among people living with HIV, underscoring the importance of routine glycaemic monitoring in this population [18].

HCV

Chronic HCV infection also exhibits a strong relationship with dysglycaemia. Viral proteins interfere with hepatic insulin signalling, promote steatosis, and sustain systemic inflammation, all of which contribute to impaired glucose regulation. Meta-analyses in predominantly high-income countries show an increased risk of type 2 diabetes among people with chronic hepatitis C, independent of traditional metabolic risk factors [19]. Although fewer African studies exist, high seroprevalence in certain regions and low treatment coverage suggest that HCV may represent an under-recognised contributor to metabolic disease on the continent.

Tuberculosis

Tuberculosis presents a bidirectional relationship with glycaemic control. Active disease frequently manifests with stress-related hyperglycaemia, while systemic inflammation, endocrine disturbances, and possible pancreatic involvement can impair glucose metabolism in the longer term. Evidence from African and Asian studies shows that some individuals exhibit persistent dysglycaemia after tuberculosis treatment, suggesting that the infection may induce lasting metabolic effects [20,21]. The coexistence of tuberculosis with HIV further complicates metabolic trajectories.

H. pylori and Periodontal Disease

Among bacterial infections, *H. pylori* has garnered interest due to its high prevalence in many African regions. Chronic gastric inflammation may alter ghrelin and leptin production, affect nutrient absorption, and contribute to systemic inflammatory signalling [11,22]. Several African and international studies report associations between *H. pylori* infection and impaired insulin sensitivity, although findings remain inconsistent [23]. Chronic periodontal disease also contributes to systemic inflammation, and observational studies link severe periodontitis with poor glycaemic control and increased diabetes risk. Interventional studies outside Africa show that periodontal therapy can improve glycaemic indices, highlighting a potentially modifiable factor in metabolic risk [24].

Cytomegalovirus

Cytomegalovirus infection is nearly universal among adults in Africa, and emerging evidence indicates possible associations with insulin resistance and elevated glycated haemoglobin levels [25]. Proposed mechanisms include immune activation and damage to pancreatic microvasculature, but data remain limited and largely derived from small, cross-sectional studies [25].

Parasitic Infections

Parasitic infections present a complex and sometimes contradictory metabolic profile. Schistosomiasis has been associated with both protective and harmful metabolic effects, possibly reflecting differences in immune responses, infection chronicity, and host physiology [26]. Malaria, although typically acute, may have lingering metabolic effects in settings where repeated infections and chronic inflammation are common. Severe malaria episodes can cause transient disturbances in glucose regulation, and recurrent disease may influence metabolic outcomes through immune and nutritional pathways [27]. Current evidence remains preliminary and requires more rigorous investigation.

Overall, the consistency of findings for HIV, HCV, and tuberculosis contrasts with the more variable evidence for other pathogens. Nevertheless, the cumulative burden of chronic infections, high exposure prevalence, and biological plausibility suggest that infectious contributors to metabolic disease merit greater clinical and research attention in African contexts.

The major pathogens implicated in infection-related dysglycaemia in African settings, along with their proposed mechanisms, strength of evidence, and clinical implications, are summarized in Table 1.

**Table 1:** Chronic infections associated with dysglycaemia in African settings Overview of major chronic infectious exposures relevant to African populations, proposed mechanisms linking infection to impaired glucose metabolism, the relative strength of epidemiologic evidence, and key clinical implications, as discussed in this review. ART, antiretroviral therapy; HCV, hepatitis C virus; TB, tuberculosis

Pathogen	Proposed metabolic mechanisms	Strength of epidemiologic evidence	Indicative study size / data scope	Key clinical implications
HIV and antiretroviral therapy	Chronic immune activation; sustained inflammation; ART-related insulin resistance and weight gain; altered fat distribution	Strong – consistent findings across African cohort and observational studies [16–18]	Large cohorts and multiple cross-sectional studies from East, Southern, and West Africa [18,25]	Routine glycaemic screening in HIV care; careful selection and monitoring of ART regimens
Hepatitis C virus	Impaired hepatic insulin signalling; steatosis; systemic inflammation	Moderate – strong global evidence with limited African-specific studies [19]	Predominantly observational studies and meta-analyses; moderate sample sizes; limited African cohorts [19]	Consider metabolic risk assessment in chronic HCV; potential metabolic improvement after antiviral therapy
Tuberculosis	Stress-related hyperglycaemia; prolonged inflammation; possible pancreatic involvement	Moderate to strong – repeated observations across TB programmes [20,21]	Programmatic screening studies and observational cohorts, including African settings [20]	Screen for dysglycaemia at TB diagnosis and after treatment completion
Helicobacter pylori	Chronic gastric inflammation; hormonal dysregulation (ghrelin, leptin); systemic inflammatory signalling	Limited to moderate – inconsistent observational findings [23]	Mostly cross-sectional studies; variable sample sizes, including African populations [23]	Possible role for metabolic monitoring in high-prevalence settings
Periodontal disease	Systemic inflammatory burden; cytokine-mediated insulin resistance	Moderate – observational evidence with limited interventional data in Africa [24]	Observational studies and systematic reviews; small to moderate sample sizes [24]	Oral health interventions may support glycaemic control
Cytomegalovirus	Chronic immune activation; microvascular injury affecting pancreatic function	Limited – small, largely cross-sectional studies [25]	Small studies with limited geographic coverage; mostly non-African cohorts [25]	Research priority rather than routine screening
Parasitic infections (e.g., schistosomiasis, malaria)	Immune modulation; chronic inflammation; nutritional and metabolic stress	Limited and mixed – conflicting findings [26,27]	Heterogeneous observational studies; small to moderate sample sizes [26,27]	Requires further longitudinal and mechanistic research

Epidemiological evidence from Africa

Evidence from African settings supports meaningful associations between chronic infections and altered glucose metabolism, although most studies remain cross-sectional and therefore cannot establish causality. Surveys conducted in regions with high burdens of HIV, tuberculosis, HCV, *H. pylori*, and periodontal disease consistently report higher prevalence of dysglycaemia among individuals with these conditions compared to uninfected controls. For example, studies evaluating adults living with HIV in East and Southern Africa document elevated rates of impaired fasting glucose and diabetes, a pattern attributed to chronic inflammation, ART-related metabolic effects, and differences in body composition compared with HIV-negative populations [28].

In tuberculosis programmes, routine screening has revealed a high frequency of hyperglycaemia at diagnosis. While a proportion of these abnormalities reflect stress responses, follow-up studies indicate that some individuals continue to exhibit impaired glucose tolerance after treatment, suggesting more persistent metabolic disruption [29]. Similarly, evidence from hepatitis C cohorts in Africa, though fewer in number, demonstrates increased seroprevalence of the infection among patients with type 2 diabetes, supporting observations reported globally [30].

Research on parasitic infections in African contexts has produced mixed findings. One study from Ethiopia reported an inverse association between *Schistosoma mansoni* infection and metabolic risk markers, raising the possibility of immunomodulatory protection [31]. Meanwhile, data from paediatric diabetes clinics in Tanzania found a high prevalence of latent tuberculosis among children with type 1 diabetes, suggesting that infection-related immune activation may influence metabolic control in younger populations [32].

Collectively, African studies reinforce the plausibility of an infection-metabolism relationship but highlight several methodological challenges. The predominance of cross-sectional designs, heterogeneous diagnostic criteria, and limited adjustment for confounders such as nutritional status and socioeconomic factors constrain interpretation. Prospective cohorts remain scarce, and laboratory capacity for advanced metabolic or inflammatory profiling is limited in many settings. Nonetheless, the recurring co-occurrence of chronic infections and dysglycaemia across diverse populations suggests that infectious exposures may contribute more substantially to diabetes risk in Africa than is currently recognised.

Clinical and public health implications

The clinical and public health implications of infection-related metabolic disturbances are particularly important in African settings where infectious diseases and diabetes frequently coexist. Routine screening for dysglycaemia among individuals with chronic infections represents a practical and cost-effective means of improving early detection. Evidence from HIV programmes demonstrates that regular glycaemic monitoring can identify abnormalities soon after the initiation of antiretroviral therapy, a period during which the risk of insulin resistance and weight gain may increase due to immune recovery and treatment-related effects. A similar rationale applies to tuberculosis care, where screening at diagnosis and again after treatment completion helps distinguish transient stress hyperglycaemia from persistent dysglycaemia [33].

Integrated service models offer an opportunity to strengthen both infectious disease and non-communicable disease control. Many HIV and tuberculosis clinics already provide regular patient follow-up, laboratory testing, and structured counselling, making them suitable platforms for including fasting glucose or glycated haemoglobin testing. Implementing such models may also improve retention in care and reduce the missed opportunities that occur when infectious disease and diabetes services operate independently [34]. Where resources permit, combining infection control with early metabolic risk assessment may reduce the long-term burden of complications and improve overall patient outcomes.

Antimicrobial and antiviral therapies also warrant consideration. Successful hepatitis C treatment, for example, has been associated with improvements in insulin sensitivity and metabolic markers in several studies outside Africa, suggesting that treating the underlying infection may partially reverse inflammatory or hepatic mechanisms contributing to dysglycaemia [35]. In contrast, certain antiretroviral therapies can exacerbate metabolic dysfunction, underscoring the need to tailor regimens and monitor patients closely after treatment changes [15-17]. Clinicians managing tuberculosis must also account for drug interactions between rifampicin and commonly used oral diabetes medications, which may reduce therapeutic efficacy [36].

Lifestyle counselling remains essential and should be adapted to the realities of chronic infection. Nutritional guidance, physical-activity recommendations, oral-health interventions, and support for smoking cessation can improve glycaemic stability while also supporting recovery from infectious diseases. Integrating these interventions into routine care may be particularly beneficial in low-resource settings, where fragmented services and limited access to specialized NCD clinics can impede early management.

At the population level, the coexistence of high infectious disease burdens with rising diabetes rates highlights the need for coordinated policy approaches. Strengthening primary healthcare systems, improving access to diagnostic tools, and developing context-appropriate guidelines for metabolic monitoring in infectious disease programmes may help address the dual burden more effectively. Investments in training, digital record systems, and laboratory capacity will further support timely identification and long-term management of dysglycaemia in high-risk groups [37].

Research gaps and future directions

Although the evidence linking chronic infections to dysglycaemia is steadily growing, substantial research gaps remain, particularly in African contexts where both infectious and metabolic diseases are common. Most available studies are cross-sectional and therefore cannot clarify whether infections precede metabolic disturbances or merely coexist due to shared social and environmental determinants. Longitudinal cohort studies are especially limited, yet they are essential for determining whether persistent infections induce lasting changes in insulin sensitivity or β-cell function, or whether apparent associations reflect temporary metabolic responses to acute illness [38].

Mechanistic research is another major gap. Few African studies have incorporated biomarkers of inflammation, immune activation, microbial translocation, or adipokine dysregulation despite their central role in explaining how infections influence glucose metabolism. Limited laboratory infrastructure has historically constrained such work, but emerging efforts to integrate immunological and metabolic profiling into ongoing infectious disease research may help address this deficit. Similarly, little is known about how co-infections, which are common in many African settings, interact to modify metabolic pathways over time.

Standardization of diagnostic approaches also remains limited. Many studies rely on variable definitions of infection exposure, and measurements of glycaemic outcomes often differ between investigations. In some settings, anaemia and haemoglobinopathies complicate the use of glycated haemoglobin, whereas fasting glucose and oral glucose tolerance testing may be inconsistently implemented. Without harmonized diagnostic criteria, comparisons across studies remain challenging, and cumulative evidence is weakened.

Interventional studies represent another underdeveloped area. Although observational research suggests that antimicrobial or antiviral therapy may alter metabolic trajectories, such as improvements seen after successful hepatitis C treatment, few African studies have evaluated whether treating chronic infections translates into long-term metabolic benefits. Likewise, integrated care models that screen for diabetes within HIV or tuberculosis programmes show promise but require formal evaluation to determine feasibility, cost-effectiveness, and patient acceptability within resource-constrained systems [39].

Finally, the broader health-system context warrants further investigation. Many African countries lack integrated data systems that link infectious disease registries with non-communicable disease surveillance platforms. Strengthening these systems may allow better tracking of metabolic outcomes in high-risk groups and support evidence-based policy development. Without investment in data infrastructure, longitudinal research, and service integration, opportunities for early detection and prevention of infection-related dysglycaemia may be missed [39].

## Conclusions

Chronic infections appear to play a meaningful and often under-recognised role in shaping metabolic health in African populations. The strongest evidence links HIV infection and ART exposure, chronic HCV infection, and tuberculosis with dysglycaemia, while additional associations with *H. pylori*, periodontal disease, cytomegalovirus, and certain parasitic infections are emerging. Although most available data are cross-sectional, biological plausibility and consistent epidemiological signals suggest that infectious exposures may contribute to diabetes risk in settings where pathogen burden remains high.

Greater clinical recognition of the infection-metabolism relationship is needed, particularly within HIV and tuberculosis programmes that already serve large patient populations and offer regular follow-up. Integrating simple glycaemic screening into these platforms represents a feasible and cost-effective opportunity for early detection. At the same time, advances in research-especially longitudinal, mechanistic, and interventional studies-are essential to define causal pathways and identify the most effective prevention and management strategies for resource-constrained health systems. Incorporating metabolic assessment into infectious disease care, strengthening diagnostic capacity, and improving data integration may help address the intertwined epidemics of infection and diabetes across Africa. Understanding and acting on these interactions will be essential for reducing the growing burden of non-communicable disease in the region.
